# Scientometric Evaluation of Research Productivity on Diabetes from the Kingdom of Saudi Arabia over the Last Two Decades (2000-2019)

**DOI:** 10.1155/2020/1514282

**Published:** 2020-10-31

**Authors:** Zohair Jamil Gazzaz, Nadeem Shafique Butt, Nadeem Alam Zubairi, Ahmad Azam Malik

**Affiliations:** ^1^Department of Medicine, Rabigh Faculty of Medicine, King Abdulaziz University, Jeddah, Saudi Arabia; ^2^Strategic Centre to the Kingdom's Vision Realization, King Abdulaziz University, Jeddah, Saudi Arabia; ^3^Department of Family and Community Medicine, Rabigh Faculty of Medicine, King Abdulaziz University, Jeddah, Saudi Arabia; ^4^Department of Pediatrics, Rabigh Faculty of Medicine, King Abdulaziz University, Jeddah, Saudi Arabia; ^5^University Institute of Public Health, The University of Lahore, Lahore, Pakistan

## Abstract

**Materials and Methods:**

Data was extracted from the Web of Science (WoS) platform and later bibliometric analysis performed using the “R-Bibliometrix” package. A wide range of indicators was explored to measure the quantity and quality of the publications related to diabetes from KSA.

**Results:**

Saudi Arabia was 28th in rank with 2600 documents (0.83% of global share). Articles were the main document type (76%). The total number of authors was 9715 from 104 countries. Three authors showed >50 publications and >100 total citations while 2 authors showed an H-index of ≥20. The USA, UK, and Egypt were other leading contributive countries in terms of corresponding authors and total citations per country. King Saud University was the major contributing affiliation followed by King Abdulaziz University. Among 865 sources, Saudi Medical Journal was the leading and consistent source over the years. Diabetes, Diabetes Mellitus, and Type 2 Diabetes were the most frequently used keywords.

**Conclusion:**

This study provides a macroscopic overview of diabetes-related research output from KSA. Overall, similar identifications and trends of top authors were observed in terms of productivity, impact, international collaborations, and organizational affiliations. Generally, an increasing productivity trend was observed with the majority published in the last 5 to 10 years. Study findings can benefit relevant stakeholders to better understand the trends and performance of diabetes-related regional research.

## 1. Introduction

Diabetes Mellitus (DM) is considered one of the leading and rapidly growing global health issues related to both morbidity and mortality. Worldwide, more than 425 million people suffer from diabetes with a global prevalence of 8.8% among adults. Furthermore, according to the World Health Organization (WHO), diabetes has also established a role as a major cause of cardiovascular diseases, blindness, kidney diseases, and limb amputations [1, 2]. It has attained epidemic dimensions in many parts of the world. The Kingdom of Saudi Arabia (KSA) is also adversely affected with a population of around 7 million living with diabetes and a population of around 3 million living with prediabetes [3]. Notably, KSA has seen an exponential growth of diabetes in recent decades. From a prevalence of <10%, it is now among the top ten countries in the world with a higher prevalence of diabetes found to be around 23.9%. According to the International Diabetes Federation (IDF), there were 4,275,200 cases of diabetes in KSA in 2017. The Middle East and North Africa (MENA) Region has more than 55 million diabetics with an expected rise to 108 million by 2045 [4].

Changing lifestyle is one of the major factors related to this alarming progression that includes unhealthy diet, lack of exercise, overweight, and obesity. In the last few decades, many socioeconomic variations have happened in KSA along with the trend of even more cases of diabetes found in urban as compared to rural settings. The development came with noticeable deviations in population lifestyle. Eating habits showed reduced use of fruits and vegetables along with an enlarged consumption of junk food with sugar-rich and carbonated drinks. Concurrently, scientific advancements such as automobiles, elevators, and escalators contributed to the diminution of physical activities and presumably, it has execrably contributed to the rise of diabetes [5].

Worldwide, the diabetes-related economic burden is assumed to be around 673 billion USD [1]. Prominently, KSA showed an upsurge of 500% in the expenses of healthcare and diabetes management with a total healthcare budget of 180 billion SR, of which a noticeable amount of 25 billion (13.9% of total) was spent directly on the diabetic Saudi population [3]. Besides, KSA is likely to spend above 0.87 billion USD excluded from the indirect costs related to diabetes and its complications, such as disabilities, unemployment, nonattendance, less productivity, and early deaths [6].

Health services improvement is known to be dependent on healthcare research [7], which also contributes to determining and quantifying health issues along with the evaluation of used interventions [8]. The scientific literature on diabetes and various research issues is usually overwhelming, increasing the demand from researchers and other stakeholders to access the real picture [9, 10]. Additionally, the quality of care relies on evidence-based guidelines evolving from updated research [11]. Furthermore, policy decisions are also supplemented by the scientific process [7].

Interestingly, it has been reported that high-income non-Arab Middle East countries are doing better than high-income Arab Middle East countries in biomedical research [12]. A study conducted in 2014 has reported that KSA is the leading contributor in diabetes mellitus research from Middle East countries [13]. Over the last few decades, the Saudi government has been taking active steps with huge investments in academics and research and it has developed many research centers to foster academic work and research. Currently, it aims to proceed in alignment with Saudi Vision 2030 to provide the required infrastructure so as to be progressive and internationally compatible, employing knowledge-based economy in science and technology. Such initiatives have not only improved health services but have also led to an increase in the quantity of undergraduate as well as postgraduate medical institutions accompanied by an increase in scientific research output [14, 15]. Focused investments in research are considered a key part in combatting the challenges as shown by many experts and are associated with Vison 2030 for KSA [16, 17]. Despite such investments, it is hard to assess the status of the current situation and identify trends, strengths, and limitations to better plan and invest in the future. Qualitative and quantitative evaluation of publications is used to measure the scientific activities and outcomes. However, the literature on the publication activities and its assessment are scarce from KSA [14].

Assessing diabetes-related research and publication trends in KSA might be the need of the time and may serve as a key to achieve the required readiness to cope with this rising health issue. Therefore, the purpose of our study was to analyze conducted research and available literature on WoS, relevant to diabetes from KSA. Moreover, this study also explored the distribution of diabetes research publications according to time, location, and institutions besides determining the topics taken up by researchers. In this way, research performance evaluation might assist higher authorities to better understand the situation and thus facilitate in decision-making so that strategic changes can be adapted. Bibliometrics is a gateway to evaluate such proceedings and fill the knowledge gap [18, 19]. This study is aimed at assessing diabetes-related research progress over the last two decades (2000-2019) from KSA using a wide range of commonly used indicators in bibliometrics.

## 2. Materials and Methods

This was a descriptive exploratory study that is aimed at providing a snapshot to get a complete overview of diabetes-related publications from KSA. According to the World Bank, the KSA with a population of 32.94 million is considered to be among the high-income Arab countries having a gross domestic product (GDP) and gross national income (GNI) per capita of $683.8 billion and $20,080, respectively [20]. It has shown huge investments in research and development and has seen an associated increase in publications in recent years.

There are numerous databases available for researchers and academicians, such as Scopus, EBSCO, Science Direct, ProQuest, and PubMed. The most relevant and commonly used database, Web of Science (WoS), was selected along with appropriate criteria, search topics, and keywords identified from the literature, to retrieve relevant documents. Although it is a Clarivate Analytics- (formerly Thomson Reuters-) maintained platform, WoS is considered the most precise and comprehensive source for scientific exploration and appraisal with the highest quality indexing. It is also assumed to be more appropriate for evaluating the research output of different regions, authors or organizations [21, 22]. It encompasses search across salient search databases, disciplines, and document types along with more than one billion searchable cited references [23].

Plans were made for this study to use a wide range of indicators that measure the quantity and quality of the publications and provide a critical picture of national and international contributions to literature related to diabetes from KSA. King Abdul Aziz University (KAU) online library and digital resources were used to access information. This research was conducted using scientometric techniques with efforts made to assure the quality of data at both initial extraction and later processing phases. The research analyzed all published documents specifically focusing on Diabetes between 2000 and 2019 in WoS. The following search strategy was used: TITLE: (“Diabet∗” OR “DM”) Refined by ADDRESS: (Saudi∗) Timespan: 2000–2019. Indexes: SCI-EXPANDED, SSCI, A&HCI, CPCI-S, CPCI-SSH, ESCI. The use of “Diabet∗” in the above strategy means all kinds of derivatives of the Diabetes search term, such as Diabetes, Diabetes Mellitus, and Diabetic are also assessed and the records retrieved. When search was conducted using “Diabet∗” and “DM” separately from Saudi Arabia, 2592 and 38 documents were identified, respectively. All 38 documents with “DM” in the title were individually explored by two researchers independently to assess their appropriateness. Thirty-six (36) documents were found to have “Type DM I,” “Type II DM,” and/or “Diabet∗” in the title. Only two documents, an article (2016) and a meeting abstract (2006), were found to be not relevant and excluded. No language limitation was imposed. The search was done on 20th March 2020. All types of publications (total = 2600) were included. Data was extracted from WoS in plain text files and later bibliometric analysis at the author, source, and document levels was performed using the “R-Bibliometrix” package [24]. The information from the retrieved documents was analyzed using various bibliometric metrics, such as journals, publication year, authors, citation reports, institutions, and countries/regions. Two researchers (AAM and NSB) independently searched and abstracted the required information to verify the process.

## 3. Results

The total number of documents focused on diabetes research indexed in WoS from 2000 to 2019 was 314,686 from 9627 sources and >200 countries with the USA, England, China, Japan, and Germany representing around 52% and contributing 26.3%, 7.7%, 7.5%, 5.4%, and 5%, respectively. Documents were found to be related to 148 research areas and 238 WoS categories. Articles (50.5%) were found to be the leading document type followed by meeting abstracts and reviews. The total number of authors' appearances was >100,000, while the total number of group authors found was 6397 with >96% of documents not showing any groups. Around 68.5% of documents did not show any funding source, and 31% (97,668) were in the open access category. When explored for this study objective, KSA was 28th in terms of contribution with 2600 documents representing around 0.83% of the global share. Regarding the type of publications being indexed from KSA, the most common publication types in the field of diabetes research are as follows: articles, representing around 76% (1979); meeting abstracts, representing around 10.7% (278); and review papers, representing around 7.7% (201).

The summary of the study is shown in Table 1, with 2600 documents published from KSA (for the period of 2000-2019) extracted from WoS with 865 sources (9.3% of the total sources). Around 58% of the documents did not show any funding source, and 5% (1233) of the documents were in the open access category. Documents were found to be related to 94 research areas (63% of total) and 132 WoS categories (55.5% of total). The total number of authors' appearances was 17400, while the total number of authors was 9715. The total number of group authors was 89 (around 1.4% of total), with >97% of documents not showing any groups. The average number of authors per article was 3.74. There were 299 (11.5%) single-authored documents with 245 authors. The average number of coauthors per document was 6.69.

Table 2 shows year-wise distribution of documents with the maximum number of publications in 2019 (436) and the minimum in 2004 (18). The initial 10 years (2000-2009) contributed 297 (11.4%) documents, while last 10 years (2010-2019) and last 5 years (2015-2019) contributed 2305 (88.6%) and 1659 (63.8%) documents, respectively.

**Figure 1:**
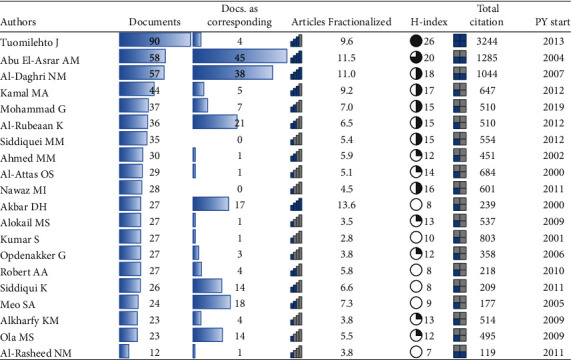
Top 20 most productive authors with authors' impact.

Figure 1 shows the 20 most productive authors with authors' impact. In total, 16 authors each have >25 publications, with 3 authors each have >50 publications, namely, Tuomilehto J (90), Abu El-Asrar AM (58), and Al-Daghri NM (57). Furthermore, the same 3 authors showed ≥1000 citations. Among the 20 most productive authors, 2 authors showed an H-index of ≥20, 8 authors showed an H-index of ≥15, and 9 authors had their starting year of publication within the last 10 years (2010-2019), as shown in Figure 1.

In diabetes research, KSA collaborated with authors from 104 countries. The top three contributing countries other than KSA were Egypt, USA, and UK with 468, 327, and 225 documents, respectively. Around 1655 (63.7%) of the corresponding authors were from KSA followed by 149, 136, and 81 from Egypt, USA, and UK, respectively. A similar trend was observed for total citations per country with 13945, 3886, 1911, and 1429 from KSA, USA, UK, and Egypt, respectively. Around 62.6% of Saudi corresponding authors published single-country publications.

**Figure 2:**
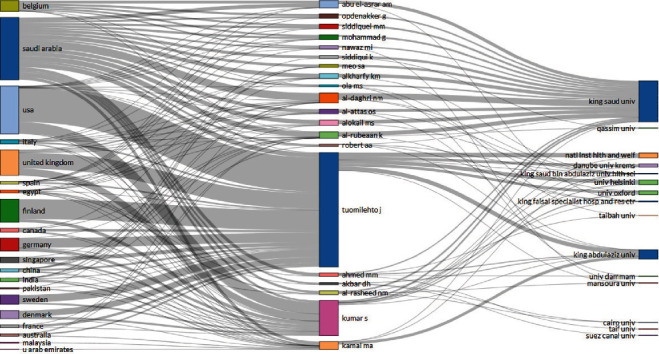
Three-field plot for the top 20 most productive country, author, and organization.

Figure 2 shows a three-field plot for the top 20 most productive countries, authors, and organizations. KSA, USA, UK, and Finland were relatively major contributing countries, while King Saud University (KSU) and King Abdulaziz University (KAU) were the major organizational contributors. The authors Tuomilehto J (mainly with KAU), Kumar S (mainly with KSU), and Al-Daghri NM (mainly with KSU) showed a maximum collaborative trend for both countries and organizations.

Table 3 shows the top 10 most frequent affiliations and funding sources. Altogether, more than 2400 institutes/organizations contributed to produce 2600 publications in the study scope. The collaborative index was 4.12. King Saud University and King Abdulaziz University were the major contributors to the total contribution from KSA on diabetes research. While Cairo University, the National Institute for Health and Welfare, and Helsinki University were the most frequent foreign affiliations. The Deanship of Scientific Research at King Saud University was the leading funding source from KSA. Endocrinology-metabolism, general internal medicine, and pharmacology-pharmacy were the most common research areas, and a similar trend was observed in WoS categories.

Based on publication output from KSA, highly cited papers were also evaluated, and the top 20 cited documents are shown in Table 4. Two (2), 4, and 25 documents are shown to have been cited >500, >300, and >100 times, respectively, showing a maximum of 558 citations. Fourteen (70%) of these 20 documents were published in the last 10 years (2010-2019). Two references were cited >100 times, namely, “Wild S, 2004, Diabetes Care (vol. 27, p. 1047)” and “Al-Nozha MM, 2004, Saudi Med J (vol. 25, p. 1603),” with a frequency of 144 and 142, respectively.

**Figure 3:**
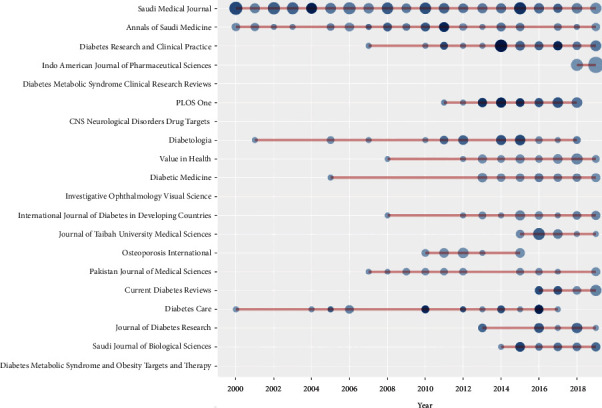
Year-wise distribution of the 20 most productive sources with citations. ^∗^Size of circle represents the number of articles, and color represents the number of citations.

Figure 3 shows the year-wise distribution of the 20 most productive sources with citations. The total number of sources is 865 with the “Saudi Medical Journal” is the leading source having 171 documents (6.6%) out of the total number of documents followed by “Annals of Saudi Medicine” and “Diabetes Research and Clinical Practice” with 47 and 43 documents, respectively.

**Figure 4:**
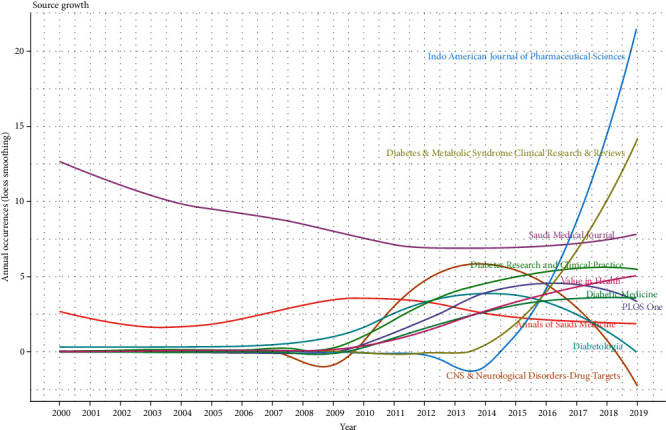
Year-wise growth of 10 most productive sources.

Figure 4 shows the year-wise growth of the 10 most productive sources. The source “Saudi Medical Journal” shows relatively consistent contributions, “Diabetes Research and Clinical Practice” shows relative growth in the last 5 years, and “Indo American Journal of Pharmaceutical Sciences” shows sudden exponential growth in the last 2 years. In contrast, two sources, “CNS & Neurological Disorders-Drug Targets” and “Diabetologia,” show a decrease in the last few years.

**Figure 5:**
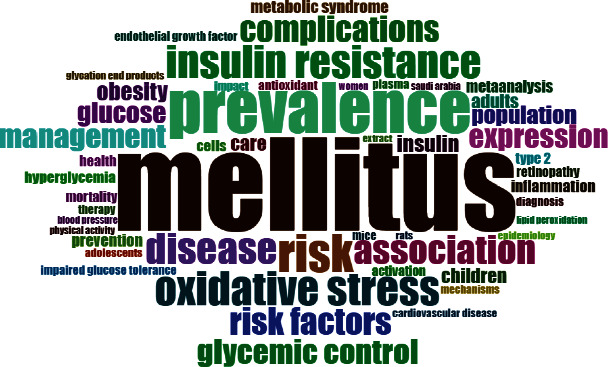
Occurrences of most frequent keywords.

**Figure 6:**
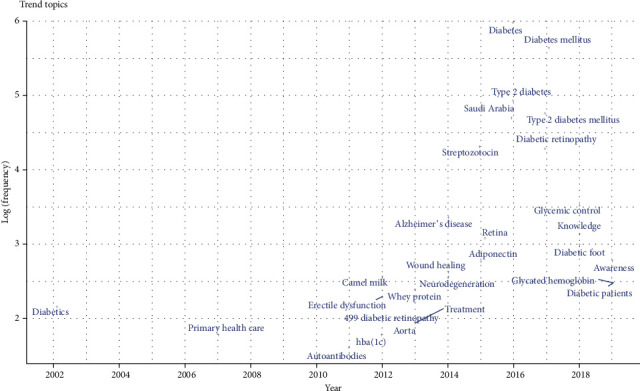
Trend of keywords by year.

In total, 4086 keywords and 4604 keywords plus (ID) were used.  Figure 4 shows the keyword map. Diabetes, Diabetes Mellitus, Type 2 Diabetes, Type 2 Diabetes Mellitus, and Saudi Arabia were the top 5 most frequent keywords used as shown in Figure 5. The trend of keywords by year is shown in Figure 6.

## 4. Discussion

One goal of Saudi Vision 2030 is to have five Saudi universities among the world's top 200 universities [25]. Additionally, KSA aims to produce an educated and skilled workforce with more efficient investments in research to meet the demands of Vision 2030 [26]. However, the literature on publication activities and its assessment are scarce from KSA [14]. The findings of this study not only tried to fill the knowledge gap and enhance academic advancement but also tried to assist in planning focused prioritizations and investments along with the avoidance of unnecessary waste in terms of resources.

This study provides a macroscopic overview with baseline information on diabetes-related research output from KSA. A bibliometric analysis was conducted for all 2600 published documents to assess the progress of diabetes-related research over the last two decades (2000-2019) from KSA using WoS as a source of data. Sources of these documents were around 9% of the total sources in the study scope while open access category documents were 5% from KSA, also relatively much lower than 31% among the total on the study scope globally. Generally, similar identifications and trends of top authors were observed in terms of productivity, impact, international collaborations, and organizational affiliations with few exceptions. An increasing trend in terms of numbers of publications was observed over the years with the majority published in the last 5 to 10 years. A resemblance was observed in global and KSA trends; for example, articles were the most frequent document type followed by meeting abstracts and reviews. Interestingly, the proportion of articles in KSA was much higher (76%) than the proportion of articles globally (50.5%) within the study scope.

Findings show that the top three productive authors (Tuomilehto J, Abu El-Asrar AM, and Al-Daghri NM) have >50 publications, and they also have ≥1000 citations and generally higher H-index, thus displaying consistency both in terms of productivity and impact. Findings suggest that in diabetes research, KSA collaborated with authors from 104 countries comprising around half of the countries contributing globally on the topic. Nearly two-thirds of the corresponding authors were from KSA. Surprisingly, the majority (62.6%) of these Saudi corresponding authors published single-country publications, which probably suggests that more effort should be put in multicountry publications. Other than KSA, Egypt, USA, and UK contributed more corresponding authors.

A relevant study conducted on diabetes mellitus research in Middle East countries found similar author collaboration trends with the UK, USA, and Germany as leading contributors [13].

Also, a similar trend was observed for total citations per country. Nearly half of the top authors showed that their starting year of publication was within the last 10 years (2010-2019). Around 70% of the 20 top-cited documents were published in the last 10 years (2010-2019).

More than 2400 institutes/organizations contributed to produce 2600 publications in the study scope. Obvious major contributors in terms of affiliations were King Saud University and King Abdulaziz University. Both universities are among the oldest and highest-ranked universities in the region. Among the top authors, Tuomilehto J showed relatively more contributions with King Abdulaziz University, while the other two authors (Abu El-Asrar AM and Al-Daghri NM) contributed with a King Saud University affiliation; interestingly, both were found to be the leading corresponding authors. Author “Al-Daghri NM” was also reported to be among the leading authors in a previous study conducted in the Middle East region [13]. An institute from Egypt (Cairo University) and 2 institutes from Finland (National Institute for Health and Welfare and Helsinki University) were the most frequent foreign affiliations. Importantly, funded publications (42%) were around 10% higher than global funding (32%) on the study topic. The Deanship of Scientific Research at King Saud University was the leading funding source, and this probably led to the major contribution from KSA. Perhaps, to achieve Saudi Vision 2030 goals, additional well-reputed local universities like King Abdulaziz University and others also need to prioritize, invest, and contribute more to study prevalent diseases like diabetes. Endocrinology-metabolism, general internal medicine, and pharmacology-pharmacy were the most common research areas, and a similar trend was observed in WoS categories showing contributions in both basic and clinical dimensions. Similar trends were observed in global productivity.

The top 3 cited documents were mainly from the Nature publishing group, and all were published in the last 6 years. Remarkably, a local journal (Saudi Medical Journal) also showed a document with >300 citations. Besides, it was also the source of one of the only 2 documents that were cited >100 times. The Saudi Medical Journal was also found to be the leading source with relatively consistent contributions over the last two decades. These findings suggest that the journal has a leading role in diabetes-related publication performance from the region. It would also be interesting to explore if such local well-reputed journals have the same standards of performance for other prevalent diseases. Diabetes, Diabetes Mellitus, Type 2 Diabetes, Type 2 Diabetes Mellitus, and Saudi Arabia were the top 5 most frequent keywords. This finding also verifies the validity of the study search strategy. Moreover, a relatively diverse and complex trend of topics was observed in the recent decade showing the evolution of more publications in various diabetes-related issues.

The analysis was based on data extracted from WoS only, and this may limit the generalizability of the findings to global diabetes research productivity. Nevertheless, findings from the analysis of the most frequent keywords strengthen the validity of the study search strategy. Documents that mentioned Deanship of Scientific Research (DSR) as a funding source with no specific information for identifying and verifying organizations/universities were categorized separately, as most of the local universities have their separate Deanship of Scientific Research units. Moreover, scarcely available literature in the study context also limited the comparison with previous decades and from other sources. Secondly, limitations in WoS along with continuous changes and updates may show different publication data to be analyzed depending upon the date of search and time frame. However, metadata from other sources and time frames might be beneficial to complement this study and provide a comprehensive context on the subject.

## 5. Conclusion

Saudi Arabia ranked 28th in terms of contribution with 2600 documents representing around 0.83% of the global share. Overall, similar trends of top authors were observed in terms of productivity, impact, international collaborations, and organizational affiliations with few exceptions. Generally, an increasing trend in terms of numbers of publications was observed over the years with the majority published in the last 5 to 10 years. Articles were found to be the most frequent document type. Findings suggest that in diabetes research, Saudi Arabia collaborated with authors from 104 countries. Nearly two-thirds of the corresponding authors were from Saudi Arabia with the majority showing single-country publications. Egypt, USA, and UK were the other leading countries contributing in terms of corresponding authors and total citations per country. King Saud University and King Abdulaziz University were major contributors. Additionally, King Saud University appeared to be the major source of funding and leading corresponding authors. The Saudi Medical Journal was the leading and consistent source over time. In conclusion, the bibliometric findings of this study can benefit relevant stakeholders and particularly researchers to better understand the performance and trends of diabetes-related research from KSA and plan with better-informed decisions with the help of these findings.

## Figures and Tables

**Table 1:** Summary table.

Period		2000-2019	
Documents	2600	Documents per author	0.268
Sources (journals, books, etc.)	865	Authors per document	3.74
Author's keywords (DE)	4086	Authors' countries	104
Open access	1233	Group authors	89
Average citations per documents	10.21	Coauthors per documents	6.69
Authors	9715	Research areas	94
Author appearances	17400	Web of Science categories	132
Authors of single-authored documents	245	Organizations	2717
Authors of multiauthored documents	9470	Funding sources	1360
Single-authored documents	299	Collaboration index	4.12

**Table 2:** Year-wise distribution of documents.

Year	*N*	Mean TC per article	Mean TC per year	Citable years	Year	*N*	Mean TC per article	Mean TC per year	Citable years
2000	25	12.3	0.6	20	2010	64	18.8	1.9	10
2001	19	13.0	0.7	19	2011	101	19.1	2.1	9
2002	30	19.1	1.1	18	2012	111	14.8	1.9	8
2003	26	16.2	1.0	17	2013	151	13.4	1.9	7
2004	18	44.3	2.8	16	2014	219	18.5	3.1	6
2005	22	20.0	1.3	15	2015	289	12.6	2.5	5
2006	36	23.6	1.7	14	2016	270	9.3	2.3	4
2007	34	16.6	1.3	13	2017	312	6.3	2.1	3
2008	40	21.8	1.8	12	2018	351	3.0	1.5	2
2009	46	24.1	2.2	11	2019	436	0.8	0.8	1

**Table 3:** Top 10 most frequent affiliations and funding sources.

Affiliations	Documents	Funding organizations	Documents
King Saud Univ	1249	Deanship of Scientific Research (DSR) at King Saud University	189
King Abdulaziz Univ	742	National Institutes of Health (NIH) USA	71
King Faisal Specialist Hosp and Res Ctr	130	United States Department of Health Human Services	71
King Khalid Univ	124	King Abdulaziz City for Science and Technology KACST	38
Cairo Univ	122	Deanship of Scientific Research (DSR)	37
Taibah Univ	110	Novo Nordisk	34
Natl Inst Hlth and Welf	107	Medical Research Council UK MRC	28
Qassim Univ	106	NIH National Institute of Diabetes Digestive Kidney Diseases NIDDK	25
Univ Helsinki	102	Deanship of Scientific Research (DSR) King Abdulaziz University Jeddah	24
Taif Univ	97	Academy of Finland	23

**Table 4:** Top 20 highly cited documents.

Document	Total citations	TC per year	Citable years
Forslund K, 2015, Nature	558	93	5
Mahajan A, 2014, Nat Genet	538	76.8	6
Fuchsberger C, 2016, Nature	421	84.2	4
Al-Nozha MM, 2004, Saudi Med J	335	19.7	16
Abdi R, 2008, Diabetes	294	22.6	12
Fiorina P, 2009, J Immunol	253	21.1	11
Flannick J, 2014, Nat Genet	250	35.7	6
Senee V, 2006, Nat Genet	196	13.1	14
Abu El-Asrar AM, 2004, Invest Ophth Vis Sci	189	11.1	16
Gaulton KJ, 2015, Nat Genet	184	30.7	5
Al-Arouj M, 2010, Diabetes Care	174	15.8	10
Moltke I, 2014, Nature	169	24.1	6
Alqurashi KA, 2011, Ann Saudi Med	168	16.8	9
Dimas AS, 2014, Diabetes	159	22.7	6
Scott RA, 2017, Diabetes	150	37.5	3
Al-Daghri NM, 2011, BMC Med	124	12.4	9
Tuomilehto J, 2013, Curr Diabetes Rep	121	15.1	7
Al-arouj M, 2005, Diabetes Care	121	7.6	15
Sacks FM, 2014, Circulation	116	16.6	6
Baothman OA, 2016, Lipids Health Dis	114	22.8	4

## Data Availability

The data used to support the findings of this study are included within the supplementary information file(s).
